# Acute-to-chronic subdural hematoma: radiographic and clinical progression from acute subdural hematoma

**DOI:** 10.1007/s10143-024-02465-2

**Published:** 2024-05-30

**Authors:** Adrian Liebert, Emily Hirschmann, Thomas Eibl, Alexander Hammer, Hans-Herbert Steiner, Karl-Michael Schebesch, Leonard Ritter

**Affiliations:** 1 Department of Neurosurgery, Paracelsus Medical University, Breslauer Straße 201, 90471 Nuremberg, Bavaria, Germany; 2 Center for Spinal and Scoliosis Surgery, Malteser Waldkrankenhaus St. Marien, Erlangen, Bavaria, Germany

**Keywords:** Acute subdural hematoma, Chronic subdural hematoma, Volume increase, Clinical deterioration, Disturbance of consciousness

## Abstract

Introduction: The pathogenesis of chronic subdural hematoma (CSDH) has not been completely understood. However, different mechanisms can result in space-occupying subdural fluid collections, one pathway can be the transformation of an original trauma-induced acute subdural hematoma (ASDH) into a CSDH. Materials and Methods: All patients with unilateral CSDH, requiring burr hole trephination between 2018 and 2023 were included. The population was distributed into an acute-to-chronic group (group A, *n* = 41) and into a conventional group (group B, *n* = 282). Clinical and radiographic parameters were analyzed. In analysis A, changes of parameters after trauma within group A are compared. In analysis B, parameters between the two groups before surgery were correlated. Results: In group A, volume and midline shift increased significantly during the progression from acute-to-chronic (*p* < 0.001, resp.). Clinical performance (modified Rankin scale, Glasgow Coma Scale) dropped significantly (*p* = 0.035, *p* < 0.001, resp.). Median time between trauma with ASDH and surgery for CSDH was 12 days. Patients treated up to the 12th day presented with larger volume of ASDH (*p* = 0.012). Before burr hole trephination, patients in group A presented with disturbance of consciousness (DOC) more often (*p* = 0.002), however less commonly with a new motor deficit (*p* = 0.014). Despite similar midline shift between the groups (*p* = 0.8), the maximal hematoma width was greater in group B (*p* < 0.001). Conclusion: If ASDH transforms to CSDH, treatment may become mandatory early due to increase in volume and midline shift. Close monitoring of these patients is crucial since DOC and rapid deterioration is common in this type of SDH.

## Introduction

Chronic subdural hematoma (CSDH) is one of the most frequent entities of intracranial hemorrhages [2, 26]. The pathogenesis of chronic subdural hematoma (CSDH) has not been completely understood. It usually develops from a mild head trauma which occurs within 6 months before clinical manifestation. However, atraumatic cases of CSDH have been reported as well [4, 7, 10, 18]. Another etiology of CSDHs is the development from an acute subdural hematoma (ASDH), which was managed conservatively initially and did not regress but liquefied and enlarged [1, 6, 9, 13, 16, 20]. Special attention should be given to this type of SDH since sudden clinical deterioration even with disturbance of consciousness (DOC) has been reported during the liquefaction phase [19]. Delayed surgery was reported to be necessary in only 6.5% of conservatively managed ASDH [3]. Risk factos which promote the development into a CSDH have been studied in multiple trials. Diabetes mellitus, use of antiplatelet or anticoagulative drugs, larger ASDH volume and midline shift are some of the risk factors for the development into a CSDH [9, 13, 16, 20]. On the one hand, identifying patients at risk is beneficial for clinical monitoring of these ASDH patients, and on the other hand, knowledge about the clinical and radiographic progression from ASDH to CSDH within the days and weeks after trauma is important for monitoring and early adequate treatment of these patients. The course of these patients has only been reported in some small case series [1, 6, 15, 19]. Also, limited data is available about potential differences of this form of CSDH and the conventional CSDH form which usually develops over a longer period of time [7, 10].

Despite the low number of reported cases with this phenomenon, special awareness should be given due to the sudden clinical deterioration. Therefore, our study aimed to analyze radiographic and clinical progression from ASDH to CSDH after trauma and risk factors for early need for burr hole trephination in this form of CSDH (acute-to-chronic SDH).

## Materials and methods

### Patient selection and study design

**Fig. 1:**
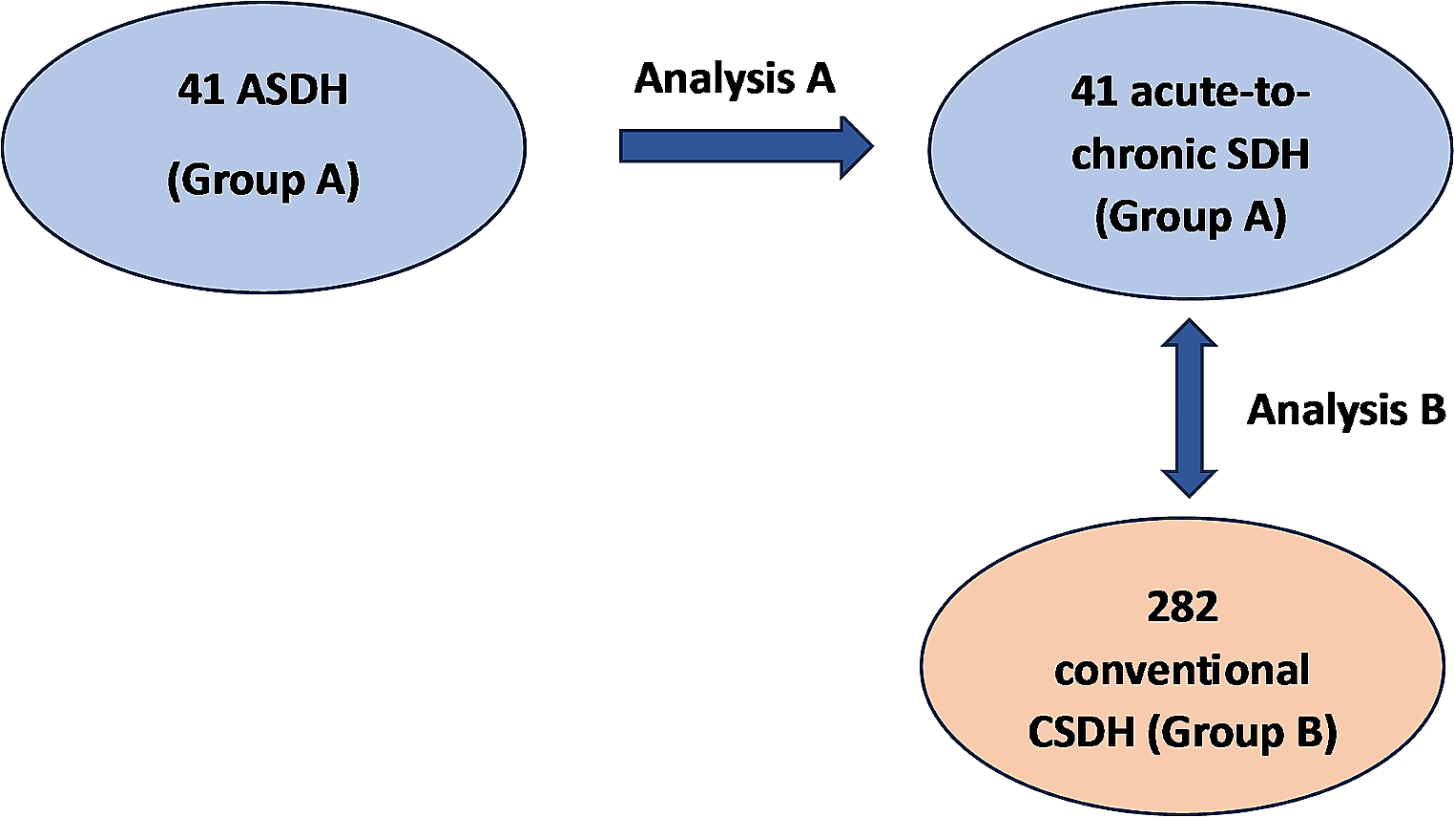
Study design including two analyses. Firstly, clinical and radiographic items within group A during the liquefaction phase were analyzed. Secondly, a comparative analysis was performed for clinical and radiographic items between the groups before burr hole trephination

A retrospective data acquisition was conducted for consecutive unilateral CSDH patients who were treated by burr hole trephination and subdural drainage in our department from January 2018 to December 2023. The study cohort was divided into an acute-to-chronic group (group A) and into a conventional group (group B). In group A, patients were included who evidently developed the CSDH from an ASDH. Patients without the demonstration of an ASDH were included in group B (Fig. 1).

The primary endpoint of this study was the volume change of SDH in group A during liquefaction phase. Secondary endpoints were time in days between trauma and surgery in group A, midline shift, maximal hematoma width, modified Rankin Scale (mRS), Glasgow Coma Scale (GCS), new motor deficit, disturbance of consciousness (DOC), epileptic seizure, death during hospital stay and recurrence within 8 weeks in group A and B. DOC in patients was defined as a drop of GCS of at least 3 points.

Exclusion criteria were the following:

- patients with recurrent or bilateral CSDHs.
- CSDH of the posterior fossa.
- patients under 18 years of age.
- CSDH patients who were treated by embolization.
- patients with ASDH who were treated by craniotomy or without the need for surgical treatment due to regressive subdural hematoma.
- patients with Glasgow Coma Scale (GCS) ≤ 8 on initial presentation with ASDH.
- atraumatic ASDH.
- patients with significant additional intracranial bleeding such as epidural hematoma, significant traumatic subarachnoid hemorrhage, traumatic intraparenchymal hematoma or with bilateral ASDH.

### Data acquisition

All data were retrospectively obtained from medical reports, radiological reports and cranial CT-imaging. The overall health status was summarized with the Charlson Comorbidity Index [5]. Radiographic data for both groups were taken from the preoperative CT scans and included the midline shift, hematoma width and locations of CSDH over the cerebral hemisphere. Location was divided into frontal, parietal, temporal and occipital location. Midline shift and hematoma width were measured on axial CT scan and the maximum was recorded. Additionally, volume of ASDH and CSDH in group A was measured by the software Visage Imaging VISAGE 7 Version 7.1.15. Maximal density of the ASDH and CSDH in Hounsfield Units (HU) was also measured as a sign of liquefaction of the hematoma. If ASDH enlarged, initial CT scan was taken with maximal volume of ASDH.

### Indication for treatment and postoperative CT scans

Indication for burr hole trephination was given for patients due to worsening of clinical symptoms and a verified sufficient liquefaction of the hematoma on CT-scan. Other patients, who were treated by craniotomy or remained with conservative treatment, were excluded from this study.

The first postoperative CT scan was conducted on the second postoperative day. A follow-up CT scan was scheduled three to four weeks after burr hole trephination. CT scan was also conducted on clinical deterioration.

Antiplatelet and anticoagulative drugs were discontinued in all patients at admission. Anticoagulative drugs were reversed with prothrombin complex and vitamin K. Antiplatelet drugs were not counteracted, neither with platelet transfusion nor desmopressin, in this patient cohort since this is only conducted in acute craniotomies in our department. Prophylaxis for thromboembolism was conducted in all patients with a low-molecular-weight heparin or unfractioned heparin in case of renal failure. Administration of prophylactic drug was started one day after trauma and discontinued for the day of surgery.

### Statistical analysis and ethics

Statistical analysis was performed with IBM SPSS-Software Version 27.

In the analysis A, clinical and radiographic data within group A was analyzed (Fig. 1). Patients clinical and radiographic parameters were compared between initial presentation with ASDH and the preoperative CT scan and presentation before surgery for CSDH. For comparison of these paired samples, the Wilcoxon rank sum test and the Wilcoxon signed-rank test were applied. For subgroup analyses within group A, the Mann-Whitney U-test was performed.

In the analysis B, clinical and radiographic parameters before burr hole trephination between group A and B were compared (Fig. 1). For this bivariate analysis, categorial variables were tested with the Pearson Chi square test or with the Fisher’s exact test. Odds ratios (OR) and 95% Confidence intervals (CI) are given for dichotomous variables which were significant. Continuous variables were first tested for normal distribution with the Kolmogorov-Smirnov test. Then the Mann-Whitney-U test was performed. The continuous variables are given as mean and standard deviation (SD).

Significance level of < 0.05 was considered statistically significant in two-sided testing.

The study was approved by the Institutional Review Board (IRB-2023-15). Due to its retrospective design, informed consent was waived.

## Results

### Patient cohort

323 patients were included in this study. 41 patients evidently developed the CSDH from an ASDH (group A) (Fig. 1). 13 subacute SDH patients were excluded from the study since they required delayed craniotomy (at least 48 h after trauma) rather than burr hole trephination.

Mean age was not different between the groups (both 77.1 years) and the median Charlson Comorbidity Index was 4 in both groups.

### Analysis A

#### Progression of radiographic and clinical parameters

**Fig. 2:**
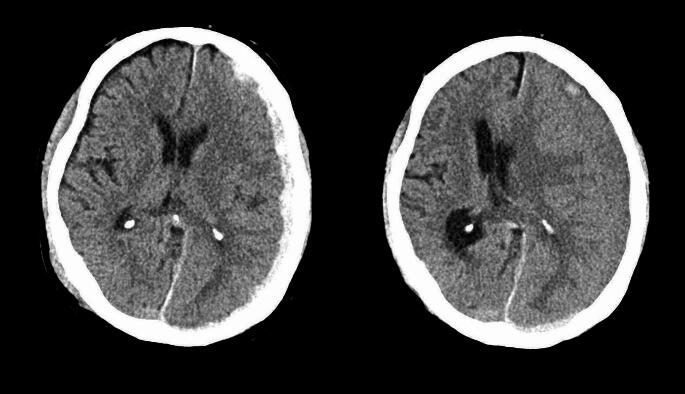
Volume and midline shift enlargement during liquefaction phase in a patient with acute-to-chronic SDH

Both, volume of subdural hematoma and midline shift during liquefaction phase increased significantly (mean 50.4 to 95.5 ml, mean 4.6 to 9.4 mm, *p* < 0.001, resp.) (Fig. 2). As expected during this period, maximal density in HU dropped significantly as well (*p* < 0.001) (Table 1). 20/41 of ASDHs spread along the entire hemisphere on the initial CT scan.

Subsequently, clinical performance summarized in the above-mentioned scores dropped significantly. Median mRS (1 to 2, *p* = 0.035) and GCS (15 to 13, *p* < 0.001) decreased during the liquefaction phase. (Table 1).

12 patients presented with DOC before surgery for CSDH, three of them with GCS < 8. Patients with DOC before surgery had a significantly higher midline shift but not a larger volume with the ASDH on the initial CT scan (5.9 vs. 4 mm, *p* = 0.025; 57.2 vs. 47.6 ml, *p* = 0.357; resp.)

Six cases in group A had to undergo second hematoma evacuation via burr hole trephination after a median time of 15 days after initial surgery.

**Table 1:** Comparison of radiographic and clinical parameter between initial presentation with ASDH and preoperative presentation for CSDH

		Initial ASDH	CSDH	*p*-value
Radiographic parameters (mean)				
	Volume (ml)	50.4 (3–127.3, SD 30.5)	95.5 (33.2–175.6, SD 34.7)	**< 0.001**
	Midline shift (mm)	4.6 (0 — 12, SD 2.7)	9.4 (2.3–20, SD 4.2)	**< 0.001**
	Maximal Density (HU)	85.6 (SD 13.4)	70.2 (SD 14.9)	**< 0.001**
Clinical performance				
	GCS (median)	15 (SD 1.2)	13 (SD 3.4)	**< 0.001**
	mRS (median)	1 (SD 1.6)	2 (SD 1.8)	**0.035**

### Factors influencing time between initial ASDH and surgery for CSDH

**Fig. 3:**
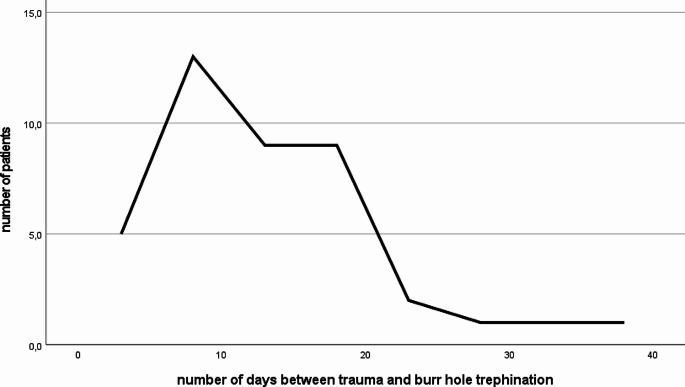
Number of patients treated via burr hole trephination over time after trauma

Median time between trauma with evidence of ASDH and surgery for CSDH was 12 days (SD 7.7, max. 38, min. 5). 23 patients (56%) were treated up to day 12. Number of patients treated over time after trauma is outlined in Fig. 3.

Patients treated up to the 12th day presented with a significantly larger initial volume of ASDH (59.5 vs. 38.8 ml, *p* = 0.012). Patients with more than 5 mm midline shift (*n* = 18) and/or with ASDH volume of more than 50 ml (*n* = 20) were treated earlier (10.5 vs. 16 days, *p* = 0.021; 11 vs. 16 days, *p* = 0.017; resp.).

Neither antiplatelet nor anticoagulative drugs had an impact on the time period from ASDH to burr hole trephination (*p* = 0.767, *p* = 0.722; resp.).

### Analysis B

In the comparative analysis between both groups, no difference in median mRS (both 2, *p* = 0.58) was evident. However, patients in group A presented with DOC significantly more often (*p* = 0.002), but less commonly with a new motor deficit (*p* = 0.014). Epileptic seizures were not more frequent in either of the groups (*p* = 1) (Table 2).

Concerning the radiographic data, frontal and parietal location were not different between the groups (both *p* = 1), but distribution of the hematoma with additional temporal and occipital locations were more common in group A (both *p* < 0.001). Hematomas with complete hemispheric distribution, over all lobes, was much more common in group A (*p* < 0.001) (Table 2).

Despite similar midline shift between the groups (8.8 vs. 8.5 mm, *p* = 0.8), the maximal hematoma width was greater in group B (15.5 vs. 22.4 mm, *p* < 0.001) (Table 2).

**Table 2:** Comparison of radiographic and clinical parameters between group A and B

Category		Group A acute-to-chronic SDH (group A)	Group B conventional CSDH (group B)	*p*-value OR 95% CI
Clinical performance				
	mRS (median)	2	2	0.58
	DOC	12/41 (29.3%)	32/282 (11.3%)	**0.002** 3 1.5-7
	New motor deficit	11/41 (26.8%)	133/282 (47.2%)	**0.014** 0.4 0.2–0.9
	Epileptic seizure	2/41 (4.9%)	15/282 (5.4%)	1
Radiographic parameters				
	Midline shift in mm (mean)	8.8 (SD 4.8)	8.5 (SD 4.6)	0.8
	Hematoma width in mm (mean)	15.5 (SD 4.2)	22.4 (SD 6.6)	**< 0.001**
	Frontal	41/41 (100%)	277/282 (98.2%)	1
	Parietal	38/41 (92.7%)	256/282 (90.8%)	1
	Temporal	36/41 (87.8%)	143/282 (50.7%)	**< 0.001** 7 2.7–18.4
	Occipital	19/41 (46.3%)	56/282 (19.9%)	**< 0.001** 3.5 1.8–6.9
	Entire hemisphere	19/41 (46.3%)	50/282 (17.7%)	**< 0.001** 4 2–8
Outcome parameters				
	Death	3/41 (7.3%)	18/282 (6.4%)	0.738
	Recurrence	6/41 (14.6%)	56/282 (19.9%)	0.427

## Discussion

In our study we analyzed the progression from ASDH to CSDH over time after trauma. The median number of days was 12 and patients with larger volume of ASDH tend to be treated earlier for CSDH. Clinical performance of patients worsened during the liquefaction course and DOC was not uncommon in this group, especially in patients with larger initial midline shift.

The path of ASDH is obvious in most cases with indication for acute evacuation via craniotomy or craniectomy in patients with lower GCS, however, in patients with initial conservative therapy and clinical monitoring, delayed craniotomy or burr hole trephination might be necessary due to expansion of the hematoma and/or clinical deterioration later on [22, 24, 28].

When awaiting liquefaction of ASDH in order to treat it with minimal approach via burr hole trephination, increase of hematoma size and midline shift while worsening clinical performance with drop of GCS should be monitored since it has been reported not only in our study [1, 6, 11]. Additionally, patients with ASDH who were initially managed conservatively were also more likely to be readmitted within 30 days due to SDH after discharge [8].

In our study the median time between trauma with ASDH and surgery for CSDH was 12 days. Kpealo et al. reported onset of symptoms after trauma with ASDH after 27.4 days in case of CSDH and 12.6 days in case of subacute SDH [19]. In a study by Kang et al., 9 patients with SDH in the subacute stage were treated via burr hole trephination and injection of urokinase in patients with increased midline shift or clinical deterioration after 10.2 days of trauma with ASDH [15]. Other studies reported similar time periods with mean number of days between 11 and 18 after trauma with ASDH and burr hole trephination for liquefied subdural hematoma [1, 6, 13].

So, our results are in accordance with the previously published literature. As outlined in Fig. 3, most patients in group A were treated between day 9 and 19. Radiographic parameters of ASDH could help to establish a schedule for follow up CT scans for conservatively managed ASDH, since in our study, patients with larger hematoma volume (esp. > 50 ml) and midline shift (esp. > 5 mm) of ASDH on the initial CT scan, presented earlier for burr hole trephination. As already known, larger hematoma size and midline shift seems to be predictive for the development of a CSDH from ASDH [9, 16]. However, the impact of ASDH volume and midline shift on outcome seems to be ambiguous still [22]. Patients with higher midline shift with ASDH also presented more common with DOC at the time of burr hole trephination in our study. 3 patients presented with a GCS < 8, whereas the other nine patients with DOC had a higher GCS.

Despite, the use of antiplatelet and anticoagulative drugs have been shown to promote chronification of ASDH, our analysis did not find a faster progression to CSDH [9, 13]. However, it must be mentioned that these drugs were discontinued and in case of coumarins antagonized. But the antiplatelet and anticoagulative drugs’ effects continue to act for a couple of days [14, 21, 25, 27]. This delayed effect did not seem to impact the time of progression in our patient cohort, though. Concerning prophylaxis for thromboembolism, early prophylaxis (< 48 h after trauma) in patients with severe SDH was reported to be safe and more effective in preventing thromboembolic events than late prophylaxis [12]. In our patient cohort, prophylaxis was started after 24 h. It must be mentioned that stable clinical and/or radiographic status should be documented before starting prophylaxis for thromboembolism. Concerning the timing of the prophylaxis, each case should be evaluated individually based on the clinical and radiographic status as well as the risk profile for thromboembolism.

When we compared this type of SDH with the conventional CSDH which develops over a couple of weeks or even months, we concluded, that acute-to-chronic SDH are symptomatic and require surgery with less hematoma size but with similar midline shift. This might be due to the faster development of CSDH from ASDH, with a median number of days of only 12 in our study, whereas conventional CSDH usually develops over a couple of weeks or even months with the formation of two membranes within the subdural space [7, 10]. This slower development of mass effect of the hematoma in conventional CSDH probably leads to a larger hematoma size without more extensive midline shift due to possible brain parenchymal compliance, especially in elderly [17]. This faster development in the acute-to-chronic entity might be also the reason for higher incidence of DOC compared to conventional type. Kpealo et al. already stressed the dangerousness of sudden drop in GCS in the liquefaction course from ASDH [19].

Motor deficit was more common in the conventional group. This might be due to higher maximal hematoma size as this factor was reported as a predictor for motor deficit before [23]. In our study, it might also be possible that DOC in the acute-to-chronic group concealed the motor deficit.

In the acute-to-chronic SDH additional temporal and occipital as well as complete distribution over one hemisphere was much more common than in the conventional group. However, this is most likely due to the wider spread over the hemisphere of the ASDH. 20/41 of ASDHs were spread along the entire hemisphere. Little is known in the literature about the distribution of the CSDH over the cerebral hemisphere and its impact. Our results indicate that acute-to-chronic SDH spreads more commonly over the entire hemisphere due to wider spread of initial ASDH.

### Strengths and limitations

Some limitations need to be addressed in this study. Due to its retrospective design a strict standardized schedule for control CT scans in group A was not achieved, clinical assessment could also be just taken from medical reports. Another limitation is the possibility that some CSDH might have also developed from ASDH in group B, even though that patients’ history was not suggestive for this.

Strengths of this study is the volume assessment via software in group A. Volume assessment was performed via a software rather than by manual calculation. The detailed analysis of radiographic and clinical parameters over time in both groups is another strength. We also report a rather high sample size of patients with the acute-to-chronic SDH form.

## Conclusion

If ASDH develops to CSDH, treatment is necessary earlier due to increase in volume and midline shift. Patients with larger ASDH volume tend to be treated even earlier. Therefore, close monitoring of these patients is crucial since sudden clinical deterioration even with DOC is not uncommon in this type of SDH.

## Data Availability

Data is provided within the manuscript and its tables.

## Abbreviations

CSDH: Chronic subdural hematoma
ASDH: Acute subdural hematoma
SDH: Subdural hematoma
GCS: Glasgow coma scale
mRS: Modified Rankin Scale
DOC: Disturbance of consciousness
